# Thyroid hormones and male reproductive system

**DOI:** 10.1186/1756-6614-6-S2-A35

**Published:** 2013-04-05

**Authors:** Krzysztof Kula

**Affiliations:** 1 Department of Andrology and Reproductive Endocrinology, Medical University of Lodz, Poland

## 

For years it was regarded that testis was unresponsive organ to thyroid hormone (TH). Recent studies have changed this view by demonstration that triiodothyronine (T3), beside follicle stimulating hormone (FSH) from pituitary, plays an essential role in testes maturation.

## Experimental hypothyroidism during sexual development

Testes size and number of spermatozoa in adulthood are determined by postnatal/pubertal proliferation of seminal tubule somatic cell constituents, the Sertoli cells. Cessation of proliferation of Sertoli cells during early development occurs concomitantly with formation of their terminally differentiated population. Transient experimental hypothyroidism in newborn rats evokes prolongation of Sertoli cell proliferation period and retards their maturation. In adulthood it results in increased Sertoli cell number, doubled final testis size in comparison to normal values, and animals produce more spermatozoa. However, prolonged hypothyroidism depresses testicular development and induces germ cell degeneration.

## The influence of T3 on seminiferous tubule development

Expression of TH receptor (TR) in rat’s Sertoli cells evolves during proliferative/maturation phase of Sertoli cells and is minimal thereafter. We have shown that T3 exerted a biphasic effect on Sertoli cell number in rats. Stimulatory influence was present before initiation of seminal tubule maturation, whereas inhibitory one appeared during initiation of pubertal development. Simultaneously, before pubertal development T3 evoked differentiation of the earliest precursors of spermatozoa, gonocytes, resulting in precocious initiation of spermatogenesis. In turn, prolonged exposition to T3 with high serum level of the hormone normalized initiation of spermatogenesis with increased cell apoptosis, together with the signs of hyperthyroidism.

## Unexpected interaction

We have shown that both T3 and FSH when given alone stimulated, whereas given in combination inhibited testes maturation. It has been shown that T3 caused disturbance of the cell cycle by controlling cyclin-dependent kinase inhibitors. This may partially explain reduced ability of Sertoli and germ cells to proliferate after combined administration of T3 and FSH.

## Clinical correlates

Hypothyroidism initiated in infancy may occur in association with macroorchidism without virilization, with impaired spermatogenesis in adulthood. When adequately treated boys with congenital hypothyroidism progress through puberty normally.

Among 3369 men aged 40-79 years overt hyperthyroidism was not frequent (0.2-0.3%) but produced erectile dysfunction in 60% of cases with no affection of sex hormone levels. Both α and β nuclear TR have been described in human corpora cavernosa cells. Hyperthyroidism impairs penile nitric oxide synthesis via direct effect and treatment of hyperthyroidism restores erectile functioning.

